# Defining data-driven subgroups of obsessive–compulsive disorder with different treatment responses based on resting-state functional connectivity

**DOI:** 10.1038/s41398-020-01045-4

**Published:** 2020-10-26

**Authors:** Seoyeon Kwak, Minah Kim, Taekwan Kim, Yoobin Kwak, Sanghoon Oh, Silvia Kyungjin Lho, Sun-Young Moon, Tae Young Lee, Jun Soo Kwon

**Affiliations:** 1 Department of Brain and Cognitive Sciences, Seoul National University College of Natural Sciences, Seoul, Republic of Korea; 2 Department of Neuropsychiatry, Seoul National University Hospital, Seoul, Republic of Korea; 3 Department of Psychiatry, Seoul National University College of Medicine, Seoul, Republic of Korea; 4 Department of Neuropsychiatry, Pusan National University Yangsan Hospital, Yangsan, Republic of Korea; 5 Institute of Human Behavioral Medicine, SNU-MRC, Seoul, Republic of Korea

**Keywords:** Neuroscience, Psychiatric disorders, Predictive markers

## Abstract

Characterization of obsessive–compulsive disorder (OCD), like other psychiatric disorders, suffers from heterogeneities in its symptoms and therapeutic responses, and identification of more homogeneous subgroups may help to resolve the heterogeneity. We aimed to identify the OCD subgroups based on resting-state functional connectivity (rsFC) and to explore their differences in treatment responses via a multivariate approach. From the resting-state functional MRI data of 107 medication-free OCD patients and 110 healthy controls (HCs), we selected rsFC features, which discriminated OCD patients from HCs via support vector machine (SVM) analyses. With the selected brain features, we subdivided OCD patients into subgroups using hierarchical clustering analyses. We identified 35 rsFC features that achieved a high sensitivity (82.74%) and specificity (76.29%) in SVM analyses. The OCD patients were subdivided into two subgroups, which did not show significant differences in their demographic and clinical backgrounds. However, one of the OCD subgroups demonstrated more impaired rsFC that was involved either within the default mode network (DMN) or between DMN brain regions and other network regions. This subgroup also showed both lower improvements in symptom severity in the 16-week follow-up visit and lower responder percentage than the other subgroup. Our results highlight that not only abnormalities within the DMN but also aberrant rsFC between the DMN and other networks may contribute to the treatment response and support the importance of these neurobiological alterations in OCD patients. We suggest that abnormalities in these connectivity may play predictive biomarkers of treatment response, and aid to build more optimal treatment strategies.

## Introduction

Individuals with obsessive–compulsive disorder (OCD) are characterized by recurrent intrusive thoughts accompanied by repetitive behaviors that provide transient relief^1–4^. However, OCD individuals present different clinical characteristics, including different comorbidities, symptom dimensions, or durations of illness. These heterogeneities complicate our understanding and treatment of the disorder. In patients with OCD, more than half of individuals are not responsive to the recommended first-line medication, such as selective serotonin reuptake inhibitors (SSRIs), with or without cognitive behavioral therapy (CBT)^5,6^. Some patients are more responsive to dopaminergic or glutamatergic agent pharmacotherapy, and ~30% are considered refractory patients^7–12^. These differences in therapeutic responses and prognosis between patients have encouraged researchers to identify homogeneous subgroups among OCD patients, which may improve treatment strategy by resolving heterogeneity. In recent decades, studies have focused on phenotype-based subtypes and the neurobiological differences and treatment responses between those subtypes^13,14^. However, these studies suffer from inconsistent results, possibly because several different dysfunctional mechanisms may present the same phenotype. Thus, more recent studies have examined subgroups based on the brain dysfunction of individuals and have shown promise in predicting treatment response^15–17^

In the psychiatric field, resting-state functional MRI (rsFMRI) is a useful neuroimaging modality to examine brain function capacity for patients because individuals often report difficulties in maintaining their attention and motivation during neurocognitive tasks^14,18–22^. Consistent evidences of aberrant DMN from the previous studies suggested that default mode network (DMN), as a prominent large-scale brain network, has a crucial role in psychological processes related to the pathophysiology of OCD^23–29^. Moreover, a recent meta-analysis study found that OCD individuals exhibited hypoconnectivity between DMN and salience network, in addition to a comprehensive dysconnectivity within DMN^30^. These findings suggest that rsfMRI-based features may effectively identify OCD subgroups with different neurobiological abnormalities.

However, most of these studies were focused on significant group differences between patients and controls. Such analyses failed to reflect the dysfunctional neural mechanisms at the individual level and underestimated brain features that may identify certain OCD subgroups. Moreover, these studies applied a standard univariate approach, which was unable to capture the dynamic interactions within and between various neural systems. For the past few years, researchers have attempted to develop analytical methods that overcome such limitations, and machine-learning analyses have been one of those methods^31,32^. Owing to its advantages, few recent OCD studies have applied machine-learning algorithms with rsFC^17,33,34^. However, these studies have one or more of the following limitations: a relatively small sample size, involvement of medicated OCD patients in the analyses, or the absence of therapeutic response information.

In this study, we aimed to explore the neural features that best discriminate OCD patients from healthy controls (HCs) and form OCD subgroups based on these rsFC features via machine learning. Then, we examined each OCD subgroup in terms of various clinical characteristics, including symptom trajectory after treatment. We hypothesized that most rsFC features that differentiate between OCD patients and HCs are those frequently reported as dysfunctional circuits, mainly included in DMN. Furthermore, we also speculated that the OCD subgroups with more deficits in those rsFC demonstrate worse at the therapeutic improvement after 16 weeks follow-up.

## Methods

### Participants

This study involved 107 medication-free OCD patients recruited from the OCD clinic at Seoul National University Hospital (SNUH) between May 2010 and October 2018. All patients with OCD fulfilled the Diagnostic and Statistical Manual of Mental Disorders-IV criteria for OCD as assessed by certified psychiatrists^35^. Of 107 medication-free patients, 45 patients were drug naive, and the remaining 62 patients had been medication free for at least 4 weeks before entering the study^36^. For each OCD patient, the Yale-Brown Obsessive–Compulsive Scale (Y-BOCS), Hamilton Rating Scale for Anxiety (HAM-A), and Hamilton Rating Scale for Depression (HAM-D) were assessed to measure the severity of obsessive–compulsive symptoms and accompanying anxiety and depressive symptoms^37–39^. The current study shares participants with a previously published study, and more details have been included in that study^30^. Prior to their participation, written informed consent was obtained from all participants after receiving a complete description of the study. This study was conducted in accordance with the Declaration of Helsinki and approved by the Institutional Review Board of SNUH.

After completing the baseline clinical assessments and MRI, the OCD patients were provided with the usual treatment, including pharmacotherapy treatment with SSRIs and CBT. In the 16-week follow-up clinical assessment, a total of 76 patients underwent clinical reevaluation with the Y-BOCS, HAM-A, and HAM-D. Responders to the initial therapeutic treatment were defined as a ≥ 35% improvement in the Y-BOCS scores^10,40–42^. Information on medications and CBT over the 16 weeks was obtained from a thorough review of medical records by a certified psychiatrist. Among the 76 OCD patients, there were 31 responders and 45 nonresponders at the follow-up visit.

### Image acquisition and preprocessing

All image data were acquired using a 3 T scanner (Magnetom Trio Siemens, Erlangen, Germany). All participants were asked to abstain from caffeine, smoking, and other stimulants before the scan. High-resolution T1-weighted anatomical images were acquired using a three-dimensional Magnetization Prepared Rapid Gradient Echo sequence with the following parameters: repetition time (TR) = 1670 ms, echo time (TE) = 1.89 ms, voxel size = 1.0 × 0.98 × 0.98 mm^3^, field of view (FoV) = 250 mm, flip angle (FA) = 9°, and 208 slices. Resting-state functional magnetic resonance imaging (rsfMRI) images were acquired with the following parameters: TR = 3500 ms, TE = 30 ms, voxel size = 1.9 × 1.9 × 3.5 mm^3^, FoV = 240 mm, FA = 9°, 35 slices, 6 minutes 58 seconds, and 116 volumes. All participants were instructed to relax their mind with eyes closed and minimize their movement to as little as possible. To ensure that participants did not fall asleep, they were reminded to stay awake immediately before the rsfMRI acquisition using a microphone, and questionnaires were provided after the scan.

The Statistical Parametric Mapping toolbox version 12 (SPM 12; Wellcome Department of Cognitive Neurology, London, UK; http://www.fil.ion.ucl.ac.uk/spm) and the CONN toolbox version 18a^43^, http://www.nitrc.org/projects/conn/) were used for preprocessing with following steps. After discarding the first four volumes due to magnetic field saturation and participants’ adaptation to the circumstance, 112 volumes were left for each subject. The remaining functional images were corrected for slice-timing discrepancies and realigned to the first scan via rigid-body alignment^44,45^. All subjects included in the present study exhibited spatial movement <2.0 mm and rotation movement <2 degree in any direction and no significant group differences in the mean head motion were observed (OCD = 0.157 ± 0.087, HCs = 0.173 ± 0.137) ^46,47^.

Using a nonlinear warping algorithm, both structural and functional images were subsequently normalized to the Montreal Neurological Institute (MNI) template and resampled to 2.0 × 2.0 × 2.0 mm^3^^48^. To increase signal-to-noise ratio, the residual images were spatially smoothed with a 4 mm full-width at half-maximum Gaussian kernel^49^. Next, smoothed images underwent the nuisance regression using a component-based noise correction method [CompCor], including principal components, which obtained from the cerebrospinal fluid and the white matter masks segmented from T1-weighted images, six head motion and their first derivatives, linear detrending, and temporal band-pass filtering (0.008–0.09 Hz) in order to clean up noise signals ^50,51^.

### fMRI connectivity feature extraction and selection

fMRI data acquisition and preprocessing are described in the Supplementary Methods section in the supplement. To generate a whole-brain correlation matrix for each individual, mean blood oxygen level-dependent time series were extracted from the 264 spherical network-defined regions of interest (ROIs) with a 10-mm diameter^52^. These ROIs had been established and extensively validated based on rsFC and meta-analysis of task fMRI studies. These ROIs belonged to the default mode (*n* = 58), salience (*n* = 18), cingulo-opercular (*n* = 14), frontoparietal (*n* = 25), dorsal and ventral attention (*n* = 20), visual and auditory (*n* = 44), somatosensory-motor (*n* = 35), and subcortical (*n* = 13) networks. ROIs that belonged to the cerebellar (*n* = 4) and undefined (*n* = 30) networks were excluded from the current study. The interregional functional connectivity strength between ROIs was calculated by computing Pearson’s bivariate correlations and transforming the correlation coefficients to the normal distribution using Fisher’s *z* transformation.

To choose the features used in the hierarchical clustering analysis, we used feature selection consisting of the following three steps: 1. selecting the lower subdiagonal features of aforementioned ROI-ROI matrix to avoid redundant connectivity with the same ROIs, 2. identifying the feature with significant group differences using *t* test with thresholding, and^3^ applying SVM analysis to find the top-ranked 35 features that best discriminate OCD patients from HCs, along with k-fold cross-validation approaches. After extracting the aforementioned ROI to ROI connectivity components (features) for each individual, we selected only the lower subdiagonal features of ROI to ROI matrix to avoid redundant connectivity with the same ROIs^53,54^. Among the 34716 rsFC features, we additionally applied an independent two-sample *t* test to individual correlation matrices to identify the rsFC features with significant differences between the OCD patients and HCs (*p* < 0.005). Each ROI was visualized using BrainNet Viewer (https://www.nitrc.org/projects/bnv/), and rsFC between two ROIs was presented with a circle map^55,56^. To represent the weight of each ROI, the node degree was calculated ^57^.

### Machine-learning analysis

To select the features with the greatest discriminative power, the SVM algorithm implemented in the Statistics Toolbox of the MATLAB software package (ver. R2017a; MathWorks Inc., Natick, MA, USA) and LIBSVM (http://www.csie.ntu.edu/tw/cjlin/libsvm/) was used. SVM is the most popular algorithm in supervised machine learning, and it performs a binary classification by maximizing the margin, allowing for an optimal separation of the training samples in complex and high-dimensional data ^53,58,59^.

In the SVM analysis using the radial basis function kernel, the hyperparameters y and C were tuned. To resolve the problems of overfitting, we applied a k-fold cross-validation approach (*k* = 5). Specifically, 217 participants were randomly split into fivefolds. Then, functional connectivities of 174 individuals were randomly used to optimize a trained model to discriminate OCD patients and HCs, whereas those of 43 individuals were used for the cross-validation of the model. Such cross-validation process was repeated 10 times with different random splits into folds. Through the process, we selected the top-ranked 35 features that best discriminate the OCD patients from the HCs. With the newly selected 35 features of 217 individuals, we conducted the fivefold cross-validation again to define the average accuracy of the 10 iterations.

**Fig. 1:**
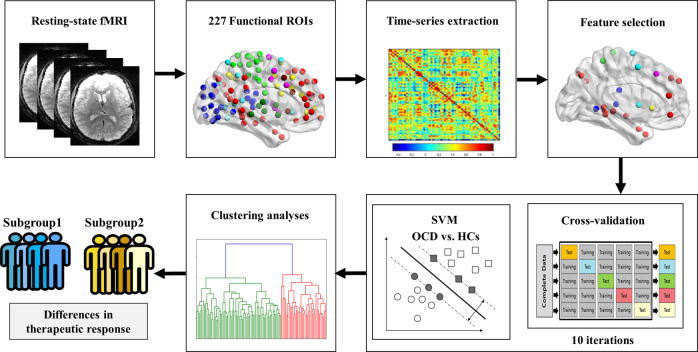
The schematic flowchart of the study. The schematic flowchart of the study, including the feature extraction as time-series extraction from 227 functional regions of interest (ROIs) from Power et al., 2011, support vector machine (SVM), and clustering analyses.

To create OCD subgroups with similar brain patterns of the 35 connectivity features, we used agglomerative hierarchical clustering implemented in a machine-learning library, scikit-learn package (v0.20.0) in Python version 2.7.6 (Python Software Foundation)^60,61^. As an unsupervised machine-learning approach, agglomerative hierarchical clustering is a technique that builds a hierarchy of clusters with a bottom–up approach by calculating a dissimilarity matrix describing the Euclidean distance between pairs of samples in the feature dimension. The optimal number of subgroups was determined using the dendrogram. The overview of the analysis involved in the study is illustrated in Fig. 1.

### Statistical analysis

To compare the demographic and clinical characteristics between groups (OCD patients vs. HCs; between OCD subgroups), independent sample *t* tests or *χ*^2^ tests were conducted using SPSS v.23.0 (IBM). To test the assumption of normality and homogeneity of variance, the Levene’s test and Shapiro–Wilk test were employed. Data variance was similar in both within and between the groups (all *ps* > 0.15). To evaluate the distribution of absences at the 16-week follow-up visit in each OCD subgroup, one-way analysis of variance was applied.

## Results

### Demographic and clinical characteristics

The demographic and clinical characteristics of individuals with OCD and HCs are summarized in Table 1. There were no statistically significant differences in the demographic backgrounds, including age, sex, handedness or years of education, between individuals with OCD and HCs.

**Table 1:** Demographic and clinical characteristics of patients with obsessive–compulsive disorder (OCD) and healthy controls (HCs).

	HCs (*n* = 110)	OCD (*n* = 107)	Statistical analysis	
	Mean (SD)	Mean (SD)	*χ*^2^/*t*	*p* value
Sex (male/female)	69/41	72/35	0.496	0.569
Handedness (right/left)	104/6	99/8	0.367	0.591
Age (years)	24.92 (6.75)	25.17 (6.57)	−0.276	0.783
Education (years)	14.35 (1.83)	14.18 (2.14)	0.631	0.529
IQ	112.89 (12.29)	110.79 (11.71)	1.292	0.198
*Baseline*				
Baseline YBOCS_T	n.a.	26.64 (6.33)		
Baseline YBOCS_O	n.a.	14.08 (2.99)		
Baseline YBOCS_C	n.a.	12.56 (4.26)		
Baseline HAM-A	n.a.	10.92 (5.98)		
Baseline HAM-D	n.a.	11.82 (6.13)		
*dYBOCS*				
Contamination	n.a.	34 (31.8%)		
Hoarding	n.a.	0 (0.0%)		
Symmetry	n.a.	21 (19.6%)		
Harm & violence	n.a.	20 (18.7%)		
Sexual & religious	n.a.	9 (8.4%)		
Miscellaneous	n.a.	23 (21.5%)		
*Comorbidity*				
None	n.a.	61 (57.0%)		
Depressive disorder	n.a.	37 (34.6%)		
Bipolar disorder	n.a.	6 (5.6%)		
Personality disorder	n.a.	3 (2.8%)		
*16-week follow-up*^a^				
16-week YBOCS_T	n.a.	18.42 (7.73)		
16-week YBOCS_O	n.a.	9.91 (4.02)		
16-week YBOCS_C	n.a.	8.49 (4.20)		
16-week HAM-A	n.a.	6.00 (4.81)		
16-week HAM-D	n.a.	6.75 (5.18)		

*dYBOCS* dimensional Yale-Brown Obsessive–Compulsive Scale, *HAM-A* Hamilton Rating Scale for Anxiety, *HAM-D* Hamilton Rating Scale for Depression, *IQ* intelligent quotient, *YBOCS_T* Yale-Brown Obsessive–Compulsive Scale total score, *YBOCS_O* Yale-Brown Obsessive–Compulsive Scale obsession score, *YBOCS_C* Yale-Brown Obsessive–Compulsive Scale compulsion score, *n.a.* not applicable.

^a^Number of missing data were 31.

### Classification between OCD patients vs HCs

The ROIs of the top 35 features that best discriminated individuals with OCD and HCs consisted of the precuneus, frontal regions, insula, parahippocampal gyrus, precentral/postcentral gyrus, supplementary motor area, angular gyrus, occipital cortex, temporal regions, and putamen. The classification accuracy of the features was 79.30%. The sensitivity and specificity were 82.74% and 76.29%, respectively. The visualization and the list of the selected features are presented in Figure S1 in the supplement.

### Clustering OCD subgroups

**Fig. 2:**
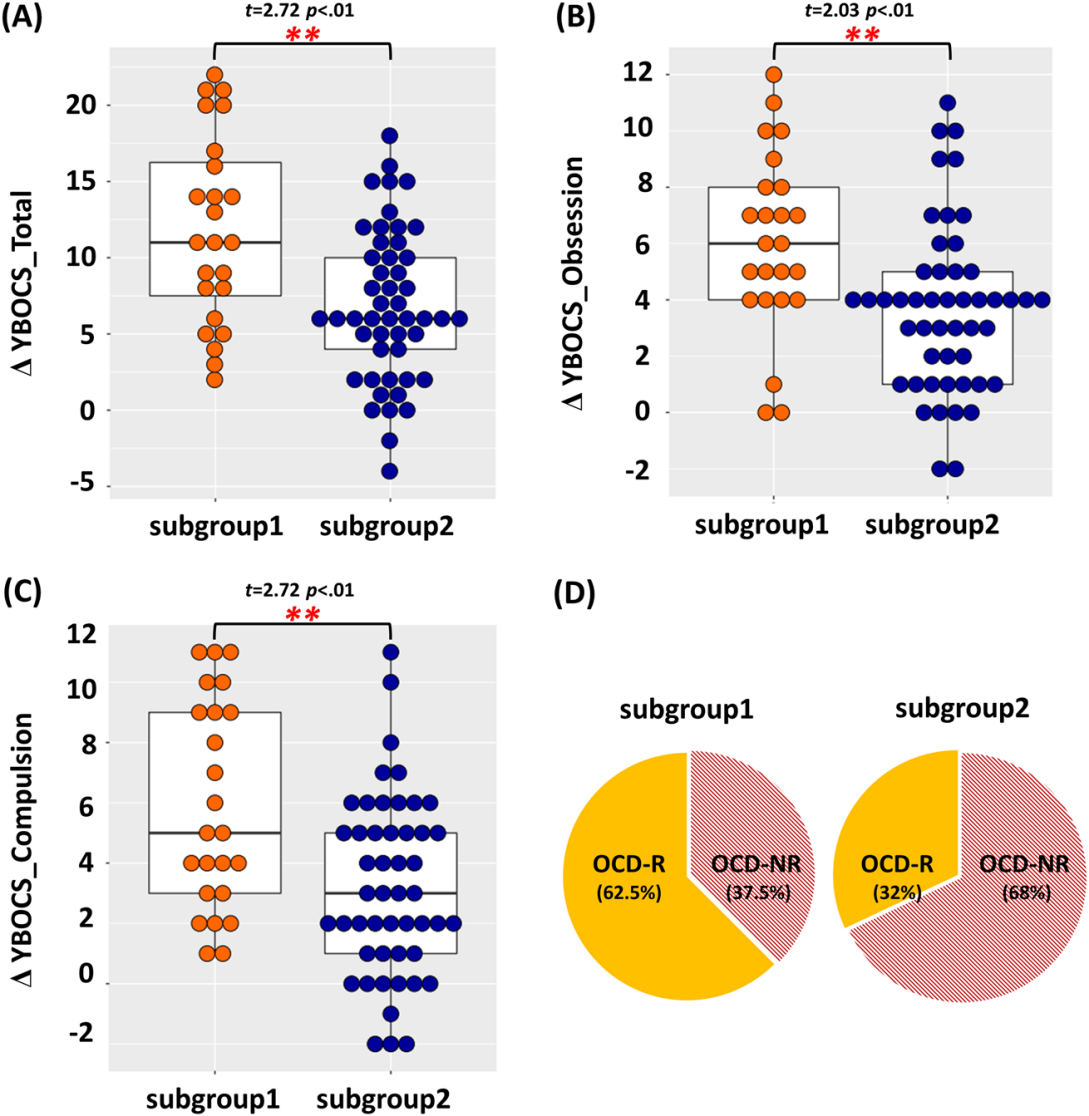
The comparision of clinical information between OCD subgroups. Boxplots indicates the comparison of the improvement Yale-Brown Obsessive–Compulsive Scale (Y-BOCS) in the **a** total scores (YBOCS_T), **b** obsession scores (YBOCS_O), and **c** compulsion (YBOCS_C) scores at the 16-week follow-up visit between the patients with obsessive–compulsive disorder (OCD) subgroups. All three scores showed significant group differences (*t* = 2.723, *p* = 0.001; *t* = 2.026, *p* = 0.006; *t* = 2.722, *p* = 0.002, respectively) **d** the pie chart demonstrated the percentage of patients with OCD showing sufficient response (OCD-R) and OCD patients did not achieve the improvement (OCD-NR) between two OCD subgroups. In the chart, subgroup 1 demonstrated significantly higher percentages of OCD-R (*χ*^2^ = 6.197, *p* = 0.013).

According to the hierarchical clustering analyses, two cluster solutions were optimal for defining relatively homogeneous subgroups, whereas the sample size in the individual clusters was sufficient for statistical power (Figure S2 in the supplement). The demographic and clinical characteristics of individuals in each OCD subgroup are summarized in Table 2. There were no significant differences in either the demographic/clinical characteristics or therapeutic interventions during the 16-week period between the two subgroups. However, the two subgroups demonstrated significant differences in clinical improvement at the 16-week follow-up clinical assessment. Subsequently, two OCD individuals were removed because they were considered outliers (greater than two standard deviations) in the Y-BOCS total score. Although two subgroups did not present any significant clinical characteristics in the baseline, they showed significant differences in the 16-week follow-up assessment scores on the Y-BOCS total and compulsions subscale and HAM-D (*t* = −2.234, *p* = 0.029; *t* = −2.095, *p* = 0.041; and *t* = −2.002, *p* = 0.049, respectively). In terms of clinical improvement, OCD subgroup 1, compared with OCD subgroup 2, demonstrated significant improvements in the Y-BOCS total scores, Y-BOCS obsession scores, Y-BOCS compulsion scores and HAM-D scores (*t* = 2.723, *p* = 0.001; *t* = 2.026, *p* = 0.006; *t* = 2.722, *p* = 0.002; and *t* = 6.197, *p* = 0.013, respectively). Considering the mean percentage of improvement from the baseline Y-BOCS total scores, the two subgroups showed significant differences (*t* = 2.295, *p* = 0.025). OCD subgroup 1 presented an improvement of 39.49 ± 21.81%, and OCD subgroup 2 demonstrated an improvement of 28.06 ± 19.85%. Moreover, 62.5% of the patients in OCD subgroup 1 were responders, whereas only 32% of patients in OCD subgroup 2 were responders (*χ*^2^ = 6.197, *p* = 0.013) (Fig. 2). The average medication dosage of each subgroup was provided in the Supplementary Results section in the supplement.

**Table 2:** Demographic and clinical characteristics of patients with obsessive–compulsive disorder (OCD) subgroups.

	OCD subgroup 1 (*n* = 38)	OCD subgroup 2 (*n* = 69)	Statistical analysis	
	Mean (SD)	Mean (SD)	*χ*^2^/*t*	*p* value
Sex (male/female)	30/8	42/27	3.638	0.084
Handedness (right/left)	33/5	66/3	5.887	0.103
Age (years)	24.79 (6.20)	25.38 (6.80)	−0.441	0.660
Education (years)	14.34 (2.16)	14.09 (2.14)	0.558	0.558
IQ	113.21 (11.65)	109.45 (11.61)	1.602	0.112
Age of onset (years)	18.79 (6.55)	18.12 (6.60)	0.506	0.614
Duration of illness (years)	6.00 (5.34)	7.26 (5.79)	−1.108	0.270
*Baseline*				
Baseline YBOCS_T	26.84 (6.39)	26.54 (6.35)	0.238	0.812
Baseline YBOCS_O	14.05 (3.39)	14.10 (2.77)	−0.081	0.936
Baseline YBOCS_C	12.79 (4.28)	12.43 (4.28)	0.410	0.682
Baseline HAM-A	9.55 (5.33)	11.68 (6.23)	−1.771	0.079
Baseline HAM-D	11.18 (6.10)	12.17 (6.17)	−0.798	0.427
*dYBOCS*			3.096	0.542
Contamination	14 (36.8%)	20 (29.0%)		
Hoarding	0 (0.0%)	0 (0.0%)		
Symmetry	9 (23.7%)	12 (17.4%)		
Harm & violence	4 (10.5%)	16 (23.2%)		
Sexual & religious	3 (7.9%)	6 (8.7%)		
Miscellaneous	8 (21.1%)	15 (21.7%)		
*Comorbidity*			1.979	0.577
None	25 (65.8%)	36 (52.2%)		
Depressive disorder	10 (26.3%)	27 (39.1%)		
Bipolar disorder	2 (4.8%)	4 (6.2%)		
Personality disorder	1 (2.4%)	2 (3.1%)		
*Medication use*				
SSRI	23 (88.5%)	45 (90.0%)	0.043	1.000
Antipsychotics	4 (15.4%)	4 (8.0%)	0.990	0.434
Mood stabilizer	1 (3.8%)	2 (4.0%)	0.001	1.000
Benzodiazepines	5 (19.2%)	10 (20.0%)	0.006	0.596
CBT	5 (19.2%)	11 (22.0%)	0.079	1.000
*16-week follow-up*^†^				
16-week YBOCS_T	15.50 (7.73)	19.20 (7.69)	−2.234	0.029*
16-week YBOCS_O	8.54 (3.22)	10.26 (4.06)	−1.970	0.054
16-week YBOCS_C	6.96 (3.51)	8.90 (4.16)	−2.095	0.041*
16-week HAM-A	5.04 (4.98)	6.52 (4.69)	−1.122	0.266
16-week HAM-D	5.12 (4.00)	7.60 (5.55)	−2.002	0.049*
*Improvement*				
Changes of YBOCS_T	10.88 (6.81)	7.08 (5.17)	2.723	0.001**
Changes of YBOC_O	5.46 (3.67)	3.84 (3.11)	2.026	0.006**
Changes of YBOCS_C	5.42 (3.72)	3.28 (2.99)	2.722	0.002**
Changes HAM-A	5.25 (4.84)	4.50 (5.16)	0.581	0.563
Changes HAM-D	6.65 (5.91)	3.74 (6.08)	1.958	0.054
Responder/non-responderª	15/9	16/34	6.197	0.013*

*CBT* cognitive behavioral therapy, *dYBOCS* dimensional Yale-Brown Obsessive–Compulsive Scale, *HAM-D* Hamilton Rating Scale for Depression, *HAM-A* Hamilton Rating Scale for Anxiety, *IQ* intelligent quotient, *YBOCS_T* Yale-Brown Obsessive–Compulsive Scale total score, YBOCS_O Yale-Brown Obsessive–Compulsive Scale obsession score, *YBOCS_C* Yale-Brown Obsessive–Compulsive Scale compulsion score.

^†^Number of follow-up missing data were 12 in group 1 and 19 in group 2; two individuals are also excluded in the analyses because they were outliers (>2 standard deviation).

ªPatients with OCD who showed ≥35% reduction in Y-BOCS total score after 16 weeks of treatment.

*The mean difference is significant at the 0.05 level.

**The mean difference is significant at the 0.01 level.

**Fig. 3:**
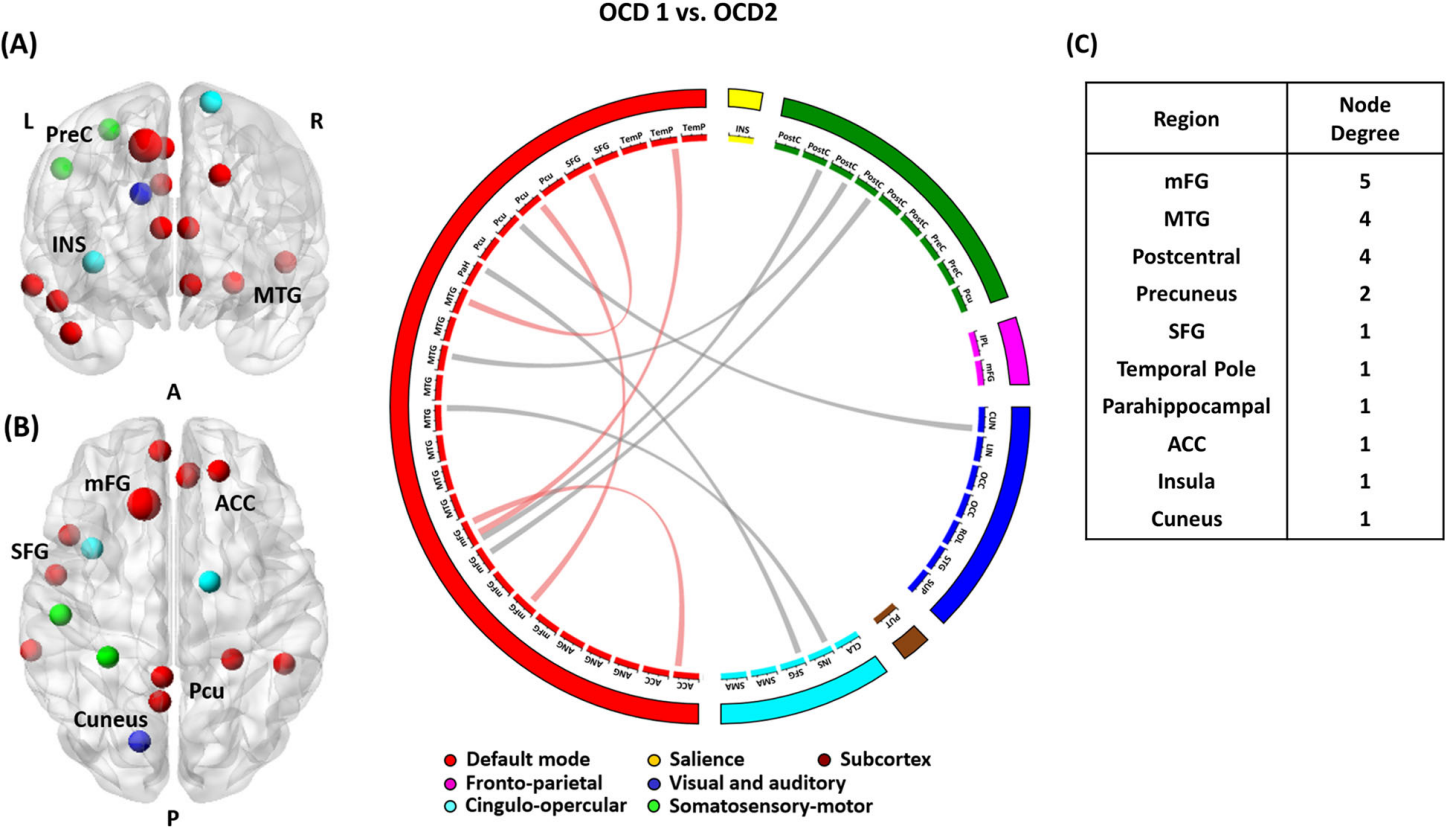
A visualization represents differences between OCD subgroup 1 (OCD1) and OCD subgroup 2 (OCD2) in the selected resting-state functional connectivity (rsFC). The rsFC is represented as a connection lines between two brain regions. Within the same network connections are colored in red, and connections between two different networks are colored in gray. **a** right hemisphere, **b** left hemisphere. **c** Node degree of each brain region. *ACC* anterior cingulate cortex, *INS* insula, *mFG* medial frontal gyrus, *MTG* middle temporal gyrus, *Pcu* precuneus, *PreC* precentral gyrus, *SFG* superior frontal gyrus.

Two subgroups also presented different patterns of brain abnormalities in the selected rsFC features. After Bonferroni corrections, the two subgroups showed significant differences in rsFC between the medial frontal gyrus (mFG) and anterior cingulate cortex (ACC), between the middle temporal gyrus (MTG) and insula, between the MTG and postcentral gyrus, between the MTG and superior frontal gyrus (SFG), between the postcentral gyrus and mFG, between the precuneus and cuneus, between the precuneus and SFG, between the SFG and mFG, and between the temporal pole and mFG (Fig. 3). Although OCD subgroup 1 demonstrated significant differences in rsFC between the occipital cortex and precuneus and rsFC between the superior temporal gyrus and mFG compared with HCs (Figure S3 in the supplement), subgroup 2 exhibited significant differences in rsFC related to the SFG, mFG, ACC, putamen, parahippocampal gyrus, angular gyrus, and lingual gyrus compared with rsFC in those regions in the HCs (Figure S4 in the supplement).

To explore the effects of presence or absence of follow-up visit on each OCD subgroups, a further analysis was performed. In the analyses, the subgroups with the absence of follow-up demonstrated a significant difference in the age of onset. Regardless of the subgroups, OCD individuals without the follow-up visit presented the higher age of onset (*F* = 3.767, *p* = 0.013). More information was provided in the Table S1 in the supplement. Another sub-analysis was also conducted for examining clinical characteristics of each subgroup, after excluding OCD individuals received CBT during the 16-week period (e.g., 15 patients received combined SSRI and CBT, 1 patient was provided CBT only), and the results were the same with the whole-group analysis. Further results of the sub-analysis were described in the Supplementary Results section and Table S2 in the supplement.

In addition, we also conducted an exploratory clustering analysis on OCD individuals with follow-up visit (*n* = 76) to confirm that the presence of follow-up visit did not affect the results of clustering analysis. Consistent with the main result, the improvement of Y-BOCS total score, obsession score, and compulsion score were significantly different, but those scores at the follow-up showed differences at the trend level. The patterns of brain abnormalities of OCD subgroups were also consistent with the main result. However, two features of OCD subgroup 2 in the exploratory analysis, rsFC between temporal Pole and Claustrum and rsFC between angular gyrus and inferior parietal lobe, were no longer significantly different compared to those of HCs. The details of this analysis are provided in the Supplementary Results section and Table S3 and Figure S5 of the supplement.

## Discussion

To the best of our knowledge, this is the first study to identify medication-free OCD subgroups with different therapeutic responses based on functional connectivity via a data-driven approach. By using two different machine-learning techniques, we defined two subgroups of OCD patients with different patterns of aberrant functional connectivity and symptom severity at 16 weeks of treatment, although there were no baseline clinical characteristic differences between the OCD subgroups. Our results also highlighted that rsFC is involved not only within the DMN network but also between the DMN and brain regions involved in other networks, and these differences in rsFC may contribute to therapeutic responses in individuals with OCD.

The 35 rsFC features, which are selected to best discriminate OCD patients from the controls via SVM results, involved brain regions consistently reported in previous studies. In particular, the mFG and temporal cortex, which had the highest node degree among the selected features, are key brain regions in the DMN. In a recent OCD machine-learning study, rsFC features within the DMN were proposed to be the best predictors of the treatment response after CBT^17^. Although that study had explored only within-network rsFC, the current results suggested that not only within-DMN rsFC but also functional connectivity between brain regions involved in the DMN were critical and that rsFC features in somatosensory-motor, visual and auditory, and cingulo-opercular networks were associated with clinical symptom severity improvement. Based on OCD subgroup 2 showing relatively increased rsFC in selected brain features, the current study suggests that this subgroup may unnecessarily waste its brain resources, and such inefficiency of resources may contribute to the therapeutic response in OCD patients.

Based on the current results, we speculate that the preservation of rsFC of DMN regions may be associated with the efficacy of pharmacotherapy or CBT. One possible explanation is related to the function of the DMN. Considering its major role in self-referential processing, abnormal functions in DMN regions may cause preoccupation of self-oriented intrusive obsessive thoughts or dysfunctional evaluations of one’s own behavioral performance^62,63^. From the neurocognitive perspective, OCD subgroup 2 may have more neurocognitive deficits in self-referential processing, especially in judgments related to one’s own characteristics^64^. Neurocognitive performance is known to be specifically related to dorsomedial prefrontal DMN subsystems, mainly involving the mFG and temporal pole^65^. As only a few OCD neurocognitive studies have explored dysfunction in self-referential processing, future studies are necessary to verify the relationship between self-referential function and treatment response in patients with OCD. Another possible explanation is the underlying mechanisms of therapeutic interventions. Indeed, SSRIs and CBT are popular first-line interventions not only for OCD but also for various anxiety disorders and depressive disorders. In anxiety or depressive disorder studies with SSRI or CBT interventions, many studies have found that baseline frontotemporal region volumes or rsFC were associated with postintervention clinical improvements^66–72^. These results may suggest possible relationships between the DMN and the efficacy of therapeutic interventions across psychiatric disorders with high comorbidities. To clarify these relationships, further studies with three patient groups, including patients with OCD, depressive disorder, and anxiety disorder, are highly encouraged.

Interestingly, the current study demonstrated that the OCD subgroups showed differences in improvements in symptom severity after therapeutic treatment, although there were no demographic or clinical characteristic differences between the subgroups in their baseline assessments. By using brain-based features, the current study overcomes the limitations of phenotype-based markers. Even though the two subgroups showed no significant differences in the phenotype, there was the possibility that the same phenotype may have originated from different underlying mechanisms, or different degrees of abnormalities in several brain regions may not have been presented at the phenotype level but may have contributed to the prognosis of the disorder. Similar to other recent machine-learning studies in the medical field, the results of the current study suggested that brain connectivity-based predictors of treatment responses seem to be more successful than the previous phenotype-based predictors^73–75^. In particular, applications of the multivariate approach will result in a more predictive model, with its advantages of a more comprehensive reflection of brain complexity. Further studies on developing prediction models for individual patients based on multiple brain features are also highly suggested. This approach may provide clues for the clinical setting in which two different patients with similar demographic backgrounds and clinical characteristics show different therapeutic responses. Moreover, prospective studies that aim to prove causality between abnormal brain connectivity at baseline and therapeutic response are required to clarify these relationships. As increasing evidence suggests that data-derived subgroups of patients show better predictions in treatment outcomes than the current diagnostic system, the application of machine learning may hasten the era of precision psychiatry ^73,76,77^.

The current study exhibits some potential limitations, and a cautious interpretation of the results is warranted for the following reasons. First, validation of the result in a fully independent sample is necessary. Although we applied cross-validation analyses to avoid the overfitting issue, there could be expected cohort-specific compounding features, such as age, population of comorbid psychiatric disorders, and IQ. To construct a reliable set of rsfMRI-based biomarkers, generalization to other samples in other centers is highly recommended. Second, comorbidity status was not evaluated using the Structured Clinical Interview for the Diagnostic and Statistical Manual of Mental Disorders (SCID). The absence of clinical interview may suggest insufficient consideration of comorbidities. However, the present study included the evaluation of comorbidity status in the usual clinical setting by certified psychiatrist and the assessment of baseline anxiety and depressive symptoms to explore clinical characteristics of the patient subgroups. Third, the current study is an observational study, so the medication and its dosage across individuals varied. However, all patients were under the same treatment conditions, and the two groups showed similar therapeutic strategies, as we mentioned in the results. However, a randomized controlled trial is highly recommended in future studies to eliminate possible confounding factors from various medication strategies.

The current study suggested that not only baseline functional connectivity within the DMN but also baseline functional connectivity between the DMN and other networks were associated with future therapeutic responses in OCD patients. The application of machine-learning analyses with a multivariate approach helped to construct neurobiologically homogeneous OCD subgroups with different therapeutic response rates to the initial treatments. As phenotype-based predictive biomarkers of OCD have reported inconsistent findings, the application of a multivariate approach is encouraged to evaluate the interactions of various brain systems at the same time. Although more original and replication studies are required, the present study will shed light on the discovery of predictive biomarker in the psychiatric field.

## Supplementary information

Supplementary

## Data Availability

The code is available upon request to the corresponding author.
