# Supplementary material for: Defining data-driven subgroups of obsessive–compulsive disorder with different treatment responses based on resting-state functional connectivity
Source: Transl Psychiatry. 2020 Oct 26;10:359. doi: 10.1038/s41398-020-01045-4 (PMC7589530; doi:10.1038/s41398-020-01045-4)
Supplement: Supplementary file 1 — Supplementary [file 41398_2020_1045_MOESM1_ESM.docx]

**Supplementary Appendix**

Supplement to: **“Defining data-driven subgroups of obsessive-compulsive disorder with different treatment responses based on resting-state functional connectivity”**

**Table of Contents**

[**Supplementary Results** 2](#_Toc46035311)

[**Supplementary Table** 4](#_Toc46035312)

[**Table S1.** Demographic and clinical characteristics of patients with obsessive-compulsive disorder (OCD) subgroups, regarding the presence or absence of the 16-week follow-up (f/u) visit. 4](#_Toc46035313)

[**Table S2**. Demographic and clinical characteristics of patients with obsessive-compulsive disorder (OCD) subgroups, after excluding the patients received CBT treatment. 6](#_Toc46035314)

[**Table S3.** Demographic and clinical characteristics of patients with obsessive-compulsive disorder (OCD) new subgroups, after the additional exploratory analyses on those with 16-week follow-up (f/u) visit. 8](#_Toc46035315)

[**Supplementary Figures** 10](#_Toc46035316)

[**Figure S1.** Brain regions involved in the top 35 most contributing resting-state functional connectivity (rsFC) selected from support vector machine (SVM). 10](#_Toc46035317)

[**Figure S2.** The dendrogram depicting hierarchical clustering. The y-axis indicates the distance between clusters and colors represent the chosen two cluster solution. 11](#_Toc46035318)

[**Figure S3**. A visualization represents differences between OCD subgroup 1 (OCD1) and healthy controls (HCs) in the selected resting-state functional connectivity (rsFC). 12](#_Toc46035319)

[**Figure S4**. A visualization represents differences between OCD subgroup 2 (OCD2) and healthy controls (HCs) in the selected resting-state functional connectivity (rsFC). 13](#_Toc46035320)

[**Figure S5**. A visualization represents the results of exploratory analysis involves OCD individuals with 16-week follow-up visit in the selected resting-state functional connectivity (rsFC). 14](#_Toc46035321)

# **Supplementary Results**

*Medication Use at the 16-week follow-up assessment*

With respect to medication use at the 16-week follow-up assessment, 17 OCD patients were prescribed monotherapy with an SSRI, and 6 patients received a combination therapy with an SSRI in OCD subgroup 1. The average dosage of each medication was as follows: escitalopram, 29.67 mg/day (n=15); fluoxetine, 58.75 mg/day (n=8); risperidone, 0.50 mg/day (n=3); quetiapine, 25.00 mg/day (n=1); lamotrigine, 75.00 mg/day (n=1); and clonazepam, 0.45 mg/day (n=5). In OCD subgroup 2, 31 patients received monotherapy with an SSRI, and 13 patients were prescribed a combination therapy with an SSRI. The average medication dosages in this subgroup were escitalopram, 33.68 mg/day (n=34); fluoxetine, 43.00 mg/day (n=10); fluvoxamine, 100.00 mg/day (n=1); paroxetine, 40.00 mg/day (n=1); aripiprazole, 2.00 mg/day (n=1); risperidone, 1.33 mg/day (n=3); divalproex, 125.00 mg/day (n=1); lamotrigine, 175.00 mg/day (n=1); alprazolam, 0.50 mg/day (n=1); clonazepam, 0.45 mg/day (n=8); and lorazepam, 0.38 mg/day (n=2).

*Clinical Characteristics of OCD subgroups without those received CBT*

Among the total of 91 OCD patient who were provided only pharmacotherapy, 21 patients in the OCD subgroup1 and 39 patients in the OCD subgroup 2 attended the 16-week follow-up clinical assessment. After that, one OCD patient was additionally removed as an outlier (> 2 standard deviation). In an independent samples t-test analysis, two subgroups showed significant differences in Y-BOCS total scores, Y-BOCS, compulsion scores, and HAM-D scores at the 16-week follow-up assessment (*t* = -2.066, *p* = 0.044; *t* = -2.220, *p* = 0.031; and *t* = -2.035, *p* = 0.047, respectively). Additionally, Y-BOCS obsession score also reached differences in a trend-level (*t* = -1.763, *p* = 0.084). Regarding the clinical improvement, the OCD subgroup 1 showed significant improvements in Y-BOCS total scores, Y-BOCS obsession scores, and Y-BOCS compulsion scores, compared to the OCD subgroup 2 (*t* = 3.384, *p* = 0.001; *t* = 2.314, *p* = 0.031; and *t* = 3.711, *p* = < 0.001, respectively). While clinical scores demonstrated the same results, the mean percentage of improvement from the baseline increased in OCD subgroup 1. While OCD subgroup 2 demonstrated 29.93±19.26 percentage of mean improvement, OCD subgroup 1 showed 46.11±17.58 percentage of mean improvement. The percentage of responders in the OCD subgroup 1 was also increased almost 10%. While 73.7% patients were responders in the OCD subgroup 1, only 36% of patients were responders in the OCD subgroup 2 (*χ*²= 7.025, *p* = 0.008).

*Exploratory analysis including patients with follow-up visit*

After discarding the 31 individuals without the follow-up visit, four patients have been changed to the other subgroups in this analysis. There were no significant differences in the demographic backgrounds between two newly assigned subgroup 1 (n=28) and subgroup 2 (n=48). In the clinical improvement during the 16-week, the Y-BOCS total scores, Y-BOCS obsession scores, Y-BOCS compulsion scores demonstrated significant changes (*t* = 2.846, *p* = 0.006; *t* = 2.151, *p* = 0.035; and *t* = 2.925, *p* = 0.005, respectively). Considering the responder rate, 57.1% of OCD patients in the subgroup 1 were responders. In the subgroup 2, only 33.3% of those were responders (*χ²*= 4.113, *p* = 0.037).

# **Supplementary Table**

## **Table S1.** Demographic and clinical characteristics of patients with obsessive-compulsive disorder (OCD) subgroups, regarding the presence or absence of the 16-week follow-up (f/u) visit.

|  | OCD subgroup 1 f/u-Y (n=26) | OCD subgroup 1 f/u-N (n=12) | OCD subgroup 2 f/u-Y (n=50) | OCD subgroup 2 f/u-N (n=19) | Statistical analysis for all 4 subgroups | | Statistical analysis for 2 subgroups with f/u-Y | |
| --- | --- | --- | --- | --- | --- | --- | --- | --- |
|  |  |  |  |  |  |  |  |  |
|  | Mean (SD) | Mean (SD) | Mean (SD) | Mean (SD) | *χ² / F* | *p-value* | *χ² / F* | *p-value* |
| Sex (Male/Female) | 20/6 | 10/2 | 32/18 | 10/9 | 4.600 | 0.204 | -1.322 | 0.305 |
| Handedness (Right/Left) | 23/3 | 10/2 | 49/1 | 17/2 | 10.243 | 0.212 | -6.006 | 0.057 |
| Age (years) | 23.88 (5.83) | 26.75 (6.80) | 24.70 (7.01) | 27.16 (6.05) | 1.235 | 0.301 | -1.039 | 0.302 |
| Education (years) | 14.08 (2.19) | 14.92 (2.07) | 14.02 (1.86) | 14.26 (2.81) | 0.590 | 0.623 | 0.119 | 0.905 |
| IQ | 112.69 (10.82) | 114.33 (13.71) | 110.32 (11.60) | 107.16 (11.64) | 1.240 | 0.299 | -0.536 | 0.593 |
| Age of onset | 17.65 (6.10) | 21.25 (7.07) | 16.76 (6.52) | 21.68 (5.52) | 3.767 | .0.013* | 0.579 | 0.564 |
| Duration of illness (years) | 6.23 (5.95) | 5.50 (3.87) | 7.94 (6.37) | 5.47 (3.37) | 1.345 | 0.264 | -1.134 | 0.260 |
| Baseline |  |  |  |  |  |  |  |  |
| Baseline YBOCS_T | 27.81 (6.05) | 24.75 (6.86) | 26.28 (6.38) | 27.21 (6.38) | 0.750 | 0.525 | 0.224 | 0.823 |
| Baseline YBOCS_O | 14.69 (2.75) | 12.67 (4.27) | 14.10 (2.76) | 14.11 (2.89) | 1.270 | 0.289 | 0.020 | 0.984 |
| Baseline YBOCS_C | 13.12 (4.43) | 12.08 (4.01) | 12.18 (4.26) | 13.11 (4.40) | 0.426 | 0.735 | 0.328 | 0.744 |
| Baseline HAM-A | 10.58 (5.83) | 7.33 (3.20) | 11.10 (6.23) | 13.16 (6.13) | 2.468 | 0.066 | -0.529 | 0.598 |
| Baseline HAM-D | 11.77 (6.48) | 9.92 (5.21) | 11.34 (5.76) | 14.37 (6.82) | 1.609 | 0.192 | 0.056 | 0.955 |
| dYBOCS |  |  |  |  | 7.754 | 0.804 | -2.215 | 0.696 |
| Contamination | 8 (30.8%) | 6 (50.0%) | 12 (24.0%) | 8 (42.1%) |  |  |  |  |
| Hoarding | 0 (0.0%) | 0 (0.0%) | 0 (0.0%) | 0 (0.0%) |  |  |  |  |
| Symmetry | 6 (23.1%) | 3 (25.0%) | 9 (18.0%) | 3 (15.8%) |  |  |  |  |
| Harm & violence | 3 (11.5%) | 1 (8.3%) | 12 (24.0%) | 4 (21.1%) |  |  |  |  |
| Sexual & religious | 2 (7.7%) | 1 (8.3%) | 5 (10.0%) | 1 (5.3%) |  |  |  |  |
| Miscellaneous | 7 (26.9%) | 1 (8.3%) | 12 (24.0%) | 3 (15.8%) |  |  |  |  |
| Comorbidity |  |  |  |  | 5.600 | 0.779 | 2.423 | 0.489 |
| None | 17 (65.4%) | 8 (66.7%) | 27 (54.0%) | 9 (47.4%) |  |  |  |  |
| Depressive disorder | 7 (26.9%) | 3 (25.0%) | 19 (38.0%) | 8 (42.1%) |  |  |  |  |
| Bipolar disorder | 0 (0.0%) | 1 (8.3%) | 1 (2.0%) | 1 (5.3%) |  |  |  |  |
| Personality disorder | 2 (7.7%) | 0 (0.0%) | 3 (6.0%) | 1 (5.3%) |  |  |  |  |
| Abbreviations: dYBOCS, dimensional Yale_Brown Obsessive-Compulsive Scale; HAMA, Hamilton Rating Scale for Anxiety; HAMD, Hamilton Rating Scale for Depression; IQ, Intelligent Quotient; YBOCS_T, Yale-Brown Obsessive-Compulsive Scale total score; YBOCS_O, Yale-Brown Obsessive-Compulsive Scale obsession score; YBOCS_C, Yale-Brown Obsessive-Compulsive Scale compulsion score; n.a, not applicable | | | | | | | | |

## **Table S2**. Demographic and clinical characteristics of patients with obsessive-compulsive disorder (OCD) subgroups, after excluding the patients received CBT treatment.

|  | OCD subgroup 1 (n=33)ª | OCD subgroup 2 (n=58)ª | Statistical analysis | |
| --- | --- | --- | --- | --- |
|  |  |  |  |  |
|  | Mean (SD) | Mean (SD) | *χ² / t* | *p-value* |
| Sex (Male/Female) | 24/7 | 34/21 | 2.198 | 0.158 |
| Handedness (Right/Left) | 28/3 | 52/3 | 0.545 | 0.663 |
| Age (years) | 24.23 (6.05)… | 25.87 (6.64) | -1.140 | 0.258 |
| Education (years) | 14.13 (2.06).... | 14.07 (2.20) | 0.116 | 0.908 |
| IQ | 112.35 (11.73) | 108.58 (11.60) | 1.443 | 0.153 |
| Age of Onset (years) | 18.68 (5.79)…. | 18.55 (6.89) | 0.090 | 0.928 |
| Duration of illness (years) | 5.55 (4.84)… | 7.33 (5.89) | -1.430 | 0.156 |
| Baseline |  |  |  |  |
| Baseline YBOCS_T | 26.74 (6.53).. | 27.31 (5.85) | -0.414 | 0.68 |
| Baseline YBOCS_O | 13.77 (3.54).. | 14.36 (2.74) | -0.861 | 0.392 |
| Baseline YBOCS_C | 12.97 (3.83).. | 12.95 (3.80) | 0.026 | 0.979 |
| Baseline HAM-A | 8.94 (4.91) | 12.00 (6.20) | -2.363 | 0.02 |
| Baseline HAM-D | 11.00 (6.33).. | 12.69 (6.23) | -1.202 | 0.233 |
| dYBOCS |  |  | 3.256 | 0.516 |
| Contamination | 11 (35.5%) | 15 (27.3%). |  |  |
| Hoarding | 0 (0.0%). | 0 (0.0%). |  |  |
| Symmetry | 8 (25.8%) | 9 (16.4%) |  |  |
| Harm & violence | 4 (12.9%) | 14 (25.5%). |  |  |
| Sexual & religious | 2 (6.5%). | 6 (10.9%) |  |  |
| Miscellaneous | 6 (19.4%) | 11 (20.0%). |  |  |
| Comorbidity |  |  | 2.305 | 0.512 |
| None | 22 (71.0%) | 30 (54.5%) |  |  |
| Depressive disorder | 7 (22.6%) | 20 (36.4%) |  |  |
| Bipolar disorder | 1 (3.2%) | 2 (3.6%) |  |  |
| Personality disorder | 1 (3.2%) | 3 (5.5%) |  |  |
| Medication Use |  |  |  |  |
| SSRI | 19 (100.0%) | 36 (100.0%) | < 0.001 | 1.000 |
| Antipsychotics | 2 (10.5%) | 1 (2.8%) | 1.448 | 0.272 |
| Mood stabilizer | 1 (5.3%) | 1 (2.8%) | 0.219 | 1.000 |
| Benzodiazepines | 3 (15.8%) | 6 (16.7%) | 0.007 | 0.627 |
| 16-week follow-up^b^ |  |  |  |  |
| 16-week YBOCS_T | 15.26 (6.70) | 19.50 (7.49) | -2.066 | ..0.044* |
| 16-week YBOCS_O | ..8.32 (3.32) | 10.22 (4.04) | -1.763 | 0.084 |
| 16-week YBOCS_C | ..6.95 (3.60) | ..9.28 (3.76) | -2.220 | ..0.031* |
| 16-week HAM-A | ..4.05 (4.42) | ..6.03 (5.03) | -1.420 | 0.162 |
| 16-week HAM-D | ..4.21 (3.71) | ..7.22 (5.85) | -2.035 | ..0.047* |
| Improvement^b^ |  |  |  |  |
| Changes of YBOCS_T | 12.74 (5.66) | 7.86 (4.76) | 3.384 | ...0.001** |
| Changes of YBOC_O | ..6.16 (2.91) | 4.28 (3.04) | 2.213 | 0.031* |
| Changes of YBOCS_C | ..6.58 (3.20) | ..3.58 (2.64).. | 3.711 | < 0.001*** |
| Changes HAM-A | ..5.89 (4.97) | ..5.34 (4.88).. | .0.387 | 0.700 |
| Changes HAM-D | ..7.47 (6.55) | 4.58 (6.51) | 1.562 | 0.124 |
| Responder^c^/Non-responder^d^ | 14/5 | 13/23 | 7.025 | ...0.008** |
| Abbreviations: dYBOCS, dimensional Yale-Brown Obsessive-Compulsive Scale; HAM-D, Hamilton Rating Scale for Depression; HAM-A, Hamilton Rating Scale for Anxiety; IQ, Intelligent Quotient; YBOCS_T, Yale-Brown Obsessive-Compulsive Scale total score; YBOCS_O, Yale-Brown Obsessive-Compulsive Scale obsession score; YBOCS_C, Yale-Brown Obsessive-Compulsive Scale compulsion score. | | | | |
| ª Number of individuals received CBT was 5 in group 1 and 11 in group 2. | | | | |
| ^b^ Number of follow-up missing data was 12 in group 1 and 19 in group 2; 1 individuals are also excluded in the analyses because they were outliers ( > 2 standard deviation) | | | | |
| ^c^ Patients with OCD who showed ≥ 35% reduction in Y-BOCS total score after 16 weeks of treatment | | | | |
| ^d^ Patients with OCD who showed < 35% reduction in Y-BOCS total score after 16 weeks of treatment | | | | |
| *. The mean difference is significant at the 0.05 level. | | | | |
| **. The mean difference is significant at the 0.01 level. | | |  |  |
| ***. The mean difference is significant at the 0.001 level. | | | | |

## **Table S3.** Demographic and clinical characteristics of patients with obsessive-compulsive disorder (OCD) new subgroups, after the additional exploratory analyses on those with 16-week follow-up (f/u) visit.

|  | OCD subgroup 1 (n=28) | OCD subgroup 2 (n=48) | Statistical analysis | |
| --- | --- | --- | --- | --- |
|  |  |  |  |  |
|  | Mean (SD) | Mean (SD) | *χ² / t* | *p-value* |
| Sex (Male/Female) | 20/8 | 32/16 | 0.186 | 0.434 |
| Handedness (Right/Left) | 25/3 | 47/1 | 2.642 | 0.139 |
| Age (years) | 23.57 (5.85) | 24.92 (7.01) | -0.441 | 0.660 |
| Education (years) | 13.96 (2.30) | 14.08 (1.76) | 0.558 | 0.558 |
| IQ | ...110.82 (10.94) | ..111.31 (11.65) | 1.602 | 0.112 |
| Age of Onset (years) | 17.25 (6.05) | 16.96 (6.59) | 0.506 | 0.614 |
| Duration of illness (years) | 6.32 (6.09) | 7.96 (6.32) | -1.108 | 0.270 |
| Baseline |  |  |  |  |
| Baseline YBOCS_T | 26.03 (7.60) | 26.65 (6.41) | -0.382 | 0.703 |
| Baseline YBOCS_O | 13.73 (3.79) | 14.35 (2.65) | -0.832 | 0.408 |
| Baseline YBOCS_C | 12.30 (4.70) | 12.30 (4.40) | -0.004 | 0.997 |
| Baseline HAM-A | 10.37 (5.71) | 11.13 (6.49) | -0.526 | 0.601 |
| Baseline HAM-D | 11.07 (6.36) | 11.57 (5.94) | -0.348 | 0.729 |
| dYBOCS |  |  | 3.096 | 0.542 |
| Contamination | 10 (35.7%) | 11 (22.9%) |  |  |
| Hoarding | 0 (0.0%) | 0 (0.0%) |  |  |
| Symmetry | 6 (21.4%) | 8 (16.7%) |  |  |
| Harm & violence | 2 (7.1%) | 13 (27.1%) |  |  |
| Sexual & religious | 2 (7.1%). | 5 (10.4%) |  |  |
| Miscellaneous | 8 (28.6%) | 11 (22.9%) |  |  |
| Comorbidity |  |  | 1.979 | 0.577 |
| None | 20 (71.4%) | 25 (32.9%) |  |  |
| Depressive disorder | 6 (21.4%) | 19 (39.6%) |  |  |
| Bipolar disorder | 0 (0.0%) | 1 (2.1%) |  |  |
| Personality disorder | 2 (7.1%) | 3 (6.3%) |  |  |
| Medication Use |  |  |  |  |
| SSRI | 25 (89.3%) | 43 (89.6%) | 0.002 | 1.000 |
| Antipsychotics | 4 (14.3%) | 4 (8.3%) | 0.665 | 0.457 |
| Mood stabilizer | 1 (3.6%). | 2 (4.2%) | 0.017 | 1.000 |
| Benzodiazepines | 6 (21.4%) | 11 (23.0%) | 1.236 | 0.539 |
| CBT | 4 (14.3%) | 12 (25.0%) | 1.221 | 0.384 |
| 16-week follow-up |  |  |  |  |
| 16-week YBOCS_T | 15.65 (7.52) | 19.20 (7.69) | -1.922 | 0.058 |
| 16-week YBOCS_O | 8.62 (3.92) | 10.26 (4.06) | -1.695 | 0.094 |
| 16-week YBOCS_C | 7.12 (3.94) | 8.90 (4.16) | -1.805 | 0.075 |
| 16-week HAM-A | 4.96 (5.03) | 6.52 (4.69) | -1.281 | 0.205 |
| 16-week HAM-D | 4.96 (4.08) | 7.60 (5.55) | -2.138 | 0.036* |
| Improvement |  |  |  |  |
| Changes of YBOCS_T | 11.00 (6.61) | 7.08 (5.17) | 2.846 | 0.006* |
| Changes of YBOC_O | 5.54 (3.55) | 3.84 (3.11) | 2.151 | 0.035* |
| Changes of YBOCS_C | 5.54 (3.56) | 3.28 (2.99) | 2.925 | 0.005* |
| Changes HAM-A | 5.13 (4.89) | 4.50 (5.16) | .0.486 | 0.628 |
| Changes HAM-D | 6.50 (5.98) | 3.74 (6.08) | 1.888 | 0.063 |
| Responder/Non-responderª | 16/12 | 16/32 | 4.113 | 0.037* |
| Abbreviations: CBT, Cognitive-Behavioral Therapy; dYBOCS, dimensional Yale-Brown Obsessive-Compulsive Scale; HAM-D, Hamilton Rating Scale for Depression; HAM-A, Hamilton Rating Scale for Anxiety; IQ, Intelligent Quotient; YBOCS_T, Yale-Brown Obsessive-Compulsive Scale total score; YBOCS_O, Yale-Brown Obsessive-Compulsive Scale obsession score; YBOCS_C, Yale-Brown Obsessive-Compulsive Scale compulsion score. | | | | |
| ª Patients with OCD who showed ≥ 35% reduction in Y-BOCS total score after 16 weeks of treatment | | | | |
| *. The mean difference is significant at the 0.05 level. | | | | |
| **. The mean difference is significant at the 0.01 level. | | | | |

# **Supplementary Figures**


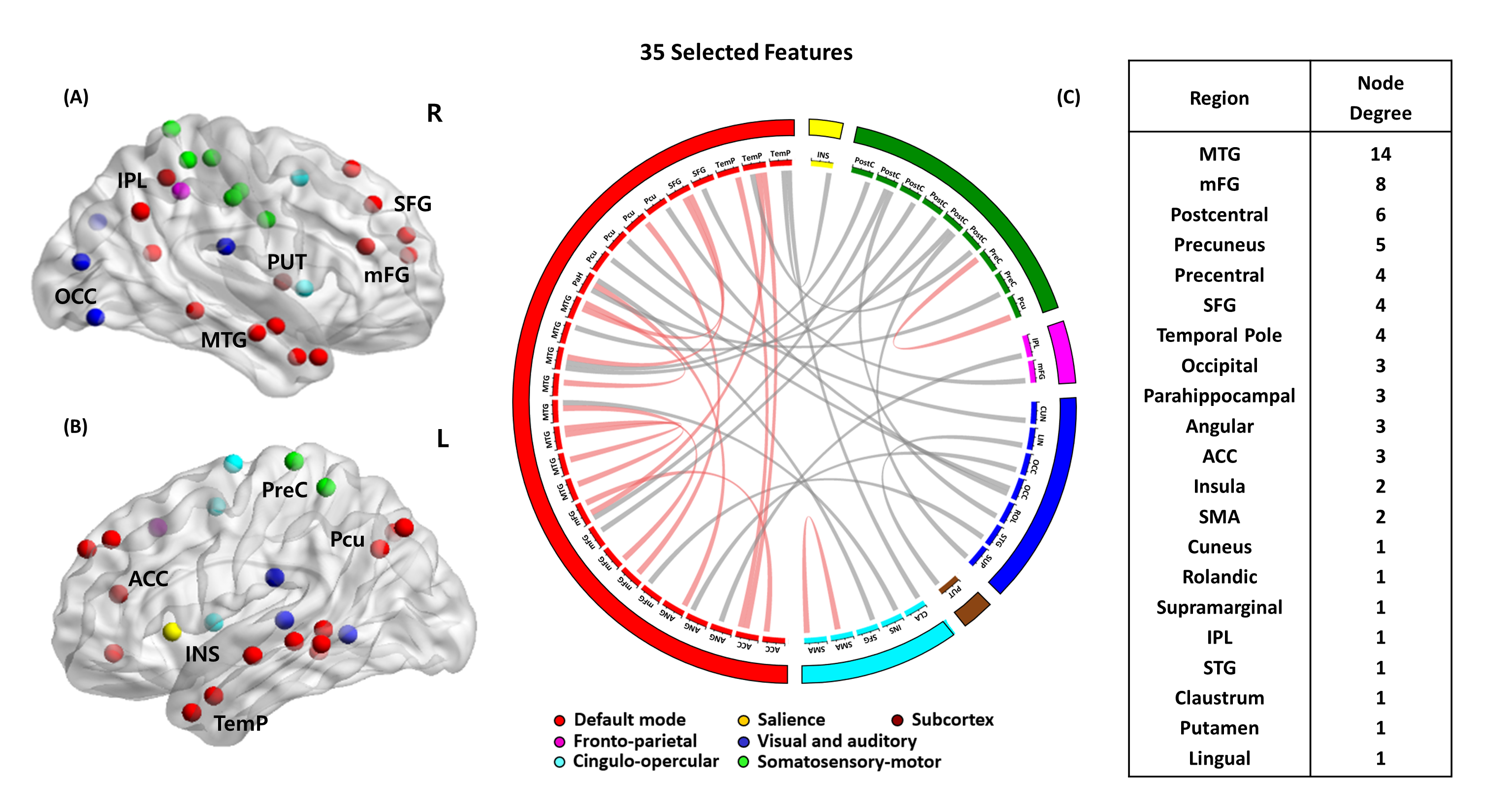


**Figure S1.** Brain regions involved in the top 35 most contributing resting-state functional connectivity (rsFC) selected from support vector machine (SVM). (A) right hemisphere, (B) left hemisphere. (C) Node degree of each brain region. Brain regions in default mode network in red; fronto-parietal network in pink, somatosensory-motor networks in green, salience network in yellow, visual and auditory network in blue, cingulo-opercular network in light blue, and subcortical network in brown. In the circle plot, the rsFC is represented as a connection lines between two brain regions. Within the same network connections are colored in red, and connections between two different networks are colored in grey. Abbreviation: ACC, anterior cingulate cortex; ANG, angular gyrus; CLA, claustrum; CUN, cuneus; INS, insula; IPL, inferior parietal lobe; LIN, lingual gyrus; mFG, medial frontal gyrus; MTG, middle temporal gyrus; PaH, parahippocampal gyrus; Pcu, precuneus; PreC, precentral gyrus; PostC, postcentral gyrus; PUT, putamen; SFG, superior frontal gyrus; ROL, rolandic sulcus; SMA, supplementary motor area; SUP, supramarginal gyrus; TemP, temporal pole; OCC, occipital cortex.


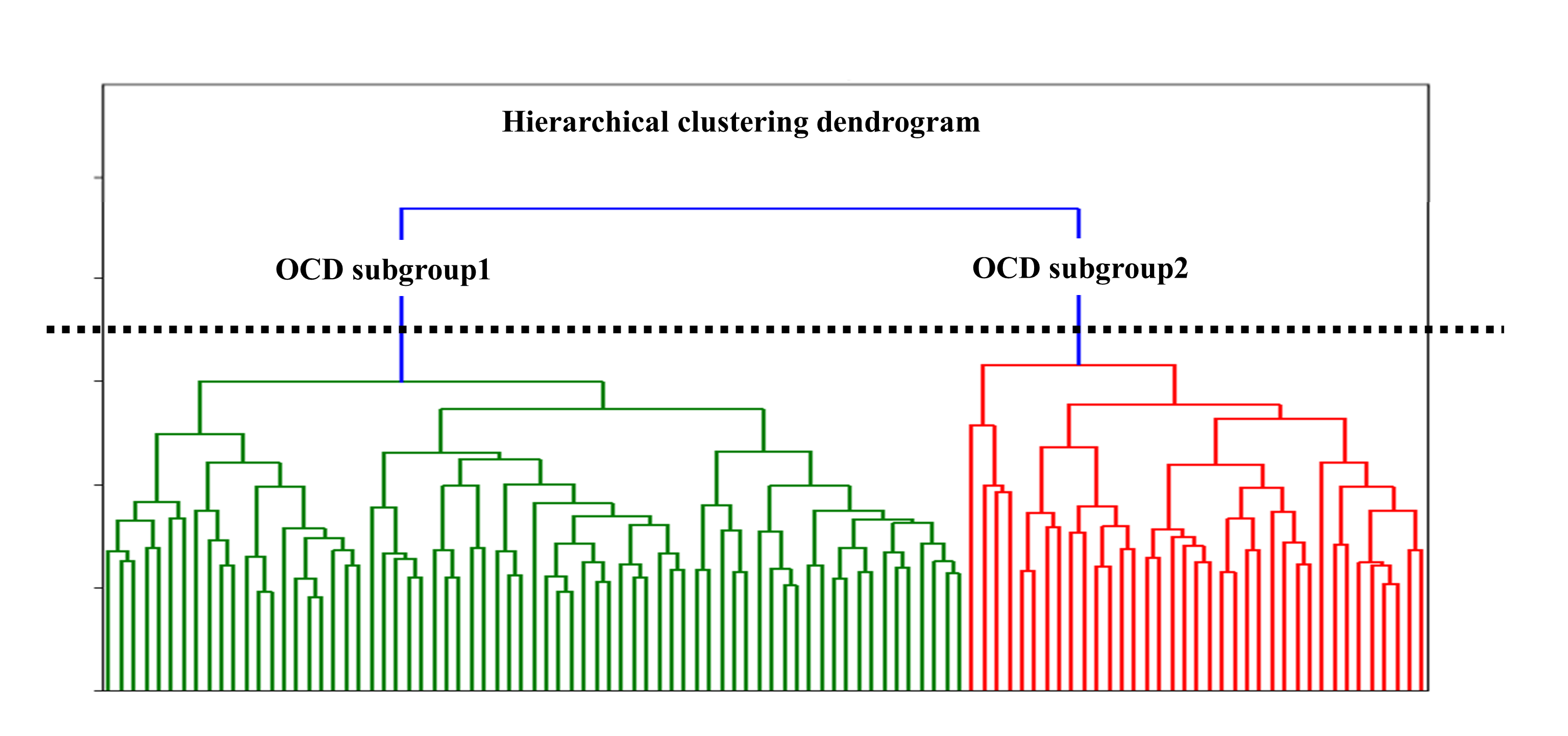


**Figure S2.** The dendrogram depicting hierarchical clustering. The y-axis indicates the distance between clusters and colors represent the chosen two cluster solution.


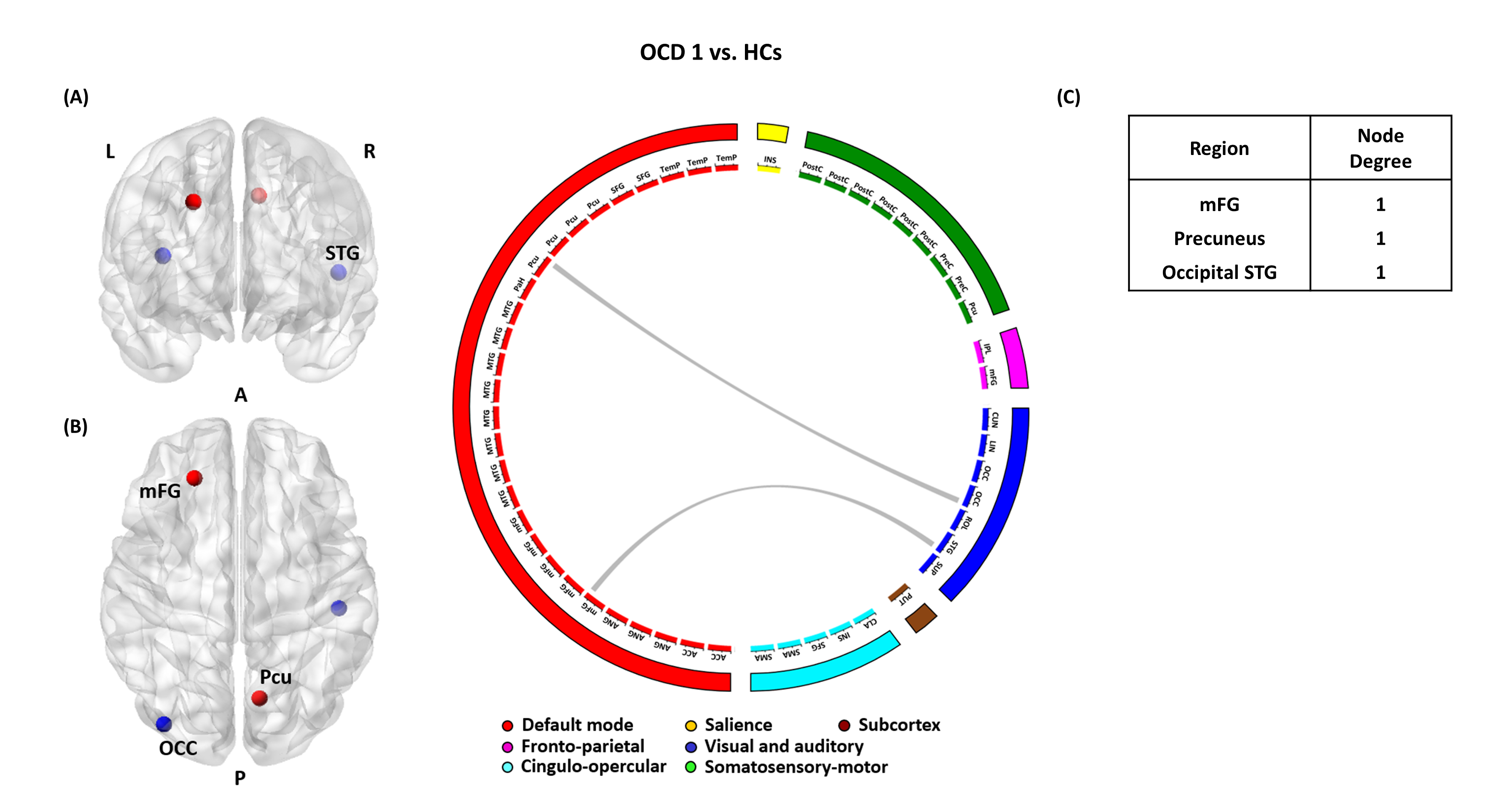


**Figure S3**. A visualization represents differences between OCD subgroup 1 (OCD1) and healthy controls (HCs) in the selected resting-state functional connectivity (rsFC). (A) right hemisphere, (B) left hemisphere. (C) Node degree of each brain region. The rsFC is represented as a connection lines between two brain regions. Within the same network connections are colored in red, and connections between two different networks are colored in grey. Abbreviation: mFG, medial frontal gyrus; Pcu, precuneus; STG, superior temporal gyrus; OCC, occipital cortex.


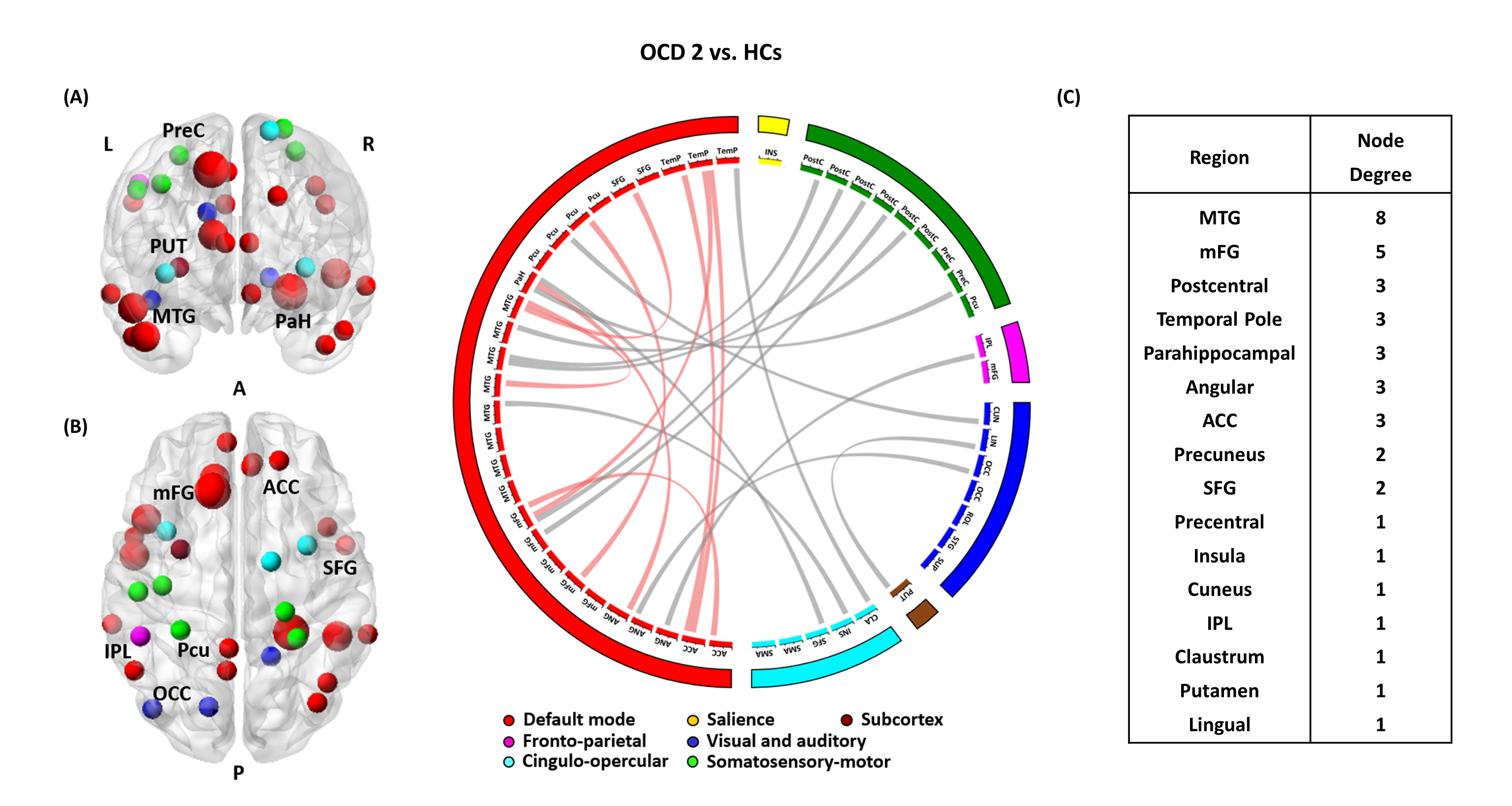


**Figure S4**. A visualization represents differences between OCD subgroup 2 (OCD2) and healthy controls (HCs) in the selected resting-state functional connectivity (rsFC). (A) right hemisphere, (B) left hemisphere. (C) Node degree of each brain region. The rsFC is represented as a connection lines between two brain regions. Within the same network connections are colored in red, and connections between two different networks are colored in grey. Abbreviation: ACC, anterior cingulate cortex; IPL, inferior parietal lobe; mFG, medial frontal gyrus; MTG, middle temporal gyrus; PaH, parahippocampal gyrus; Pcu, precuneus; PreC, precentral gyrus; PUT, putamen; SFG, superior frontal gyrus; OCC, occipital cortex.


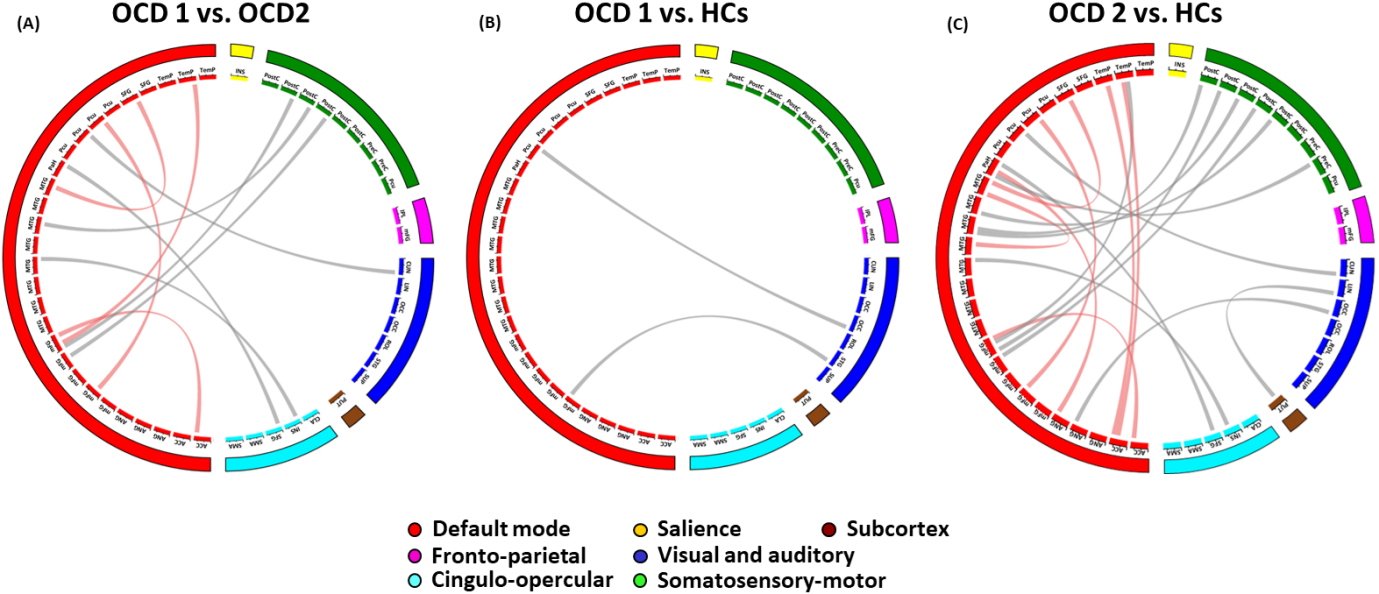


**Figure S5**. A visualization represents the results of exploratory analysis involves OCD individuals with 16-week follow-up visit in the selected resting-state functional connectivity (rsFC) between (A) OCD 1 vs. OCD 2, (B) OCD 1 vs. HCs, and (C) OCD 2 vs. HCs. The rsFC is represented as a connection lines between two brain regions. Within the same network connections are colored in red, and connections between two different networks are colored in grey. Abbreviation: ACC, anterior cingulate cortex; IPL, inferior parietal lobe; mFG, medial frontal gyrus; MTG, middle temporal gyrus; PaH, parahippocampal gyrus; Pcu, precuneus; PreC, precentral gyrus; PUT, putamen; SFG, superior frontal gyrus; OCC, occipital cortex.
